# Claudin 13, a Member of the Claudin Family Regulated in Mouse Stress Induced Erythropoiesis

**DOI:** 10.1371/journal.pone.0012667

**Published:** 2010-09-10

**Authors:** Pamela D. Thompson, Hannah Tipney, Andy Brass, Harry Noyes, Steve Kemp, Jan Naessens, May Tassabehji

**Affiliations:** 1 Cancer Immunogenetics, University of Manchester, St Mary's Hospital, Manchester, United Kingdom; 2 Center for Computational Pharmacology, University of Colorado Denver, Aurora, Colorado, United States of America; 3 School of Computer Science and Faculty of Life Sciences, University of Manchester, Manchester, United Kingdom; 4 School of Biological Sciences, University of Liverpool, Liverpool, United Kingdom; 5 International Livestock Research Institute, Nairobi, Kenya; 6 Genetic Medicine, University of Manchester, St Mary's Hospital, Manchester, United Kingdom; BMSI-A*STAR, Singapore

## Abstract

Mammals are able to rapidly produce red blood cells in response to stress. The molecular pathways used in this process are important in understanding responses to anaemia in multiple biological settings. Here we characterise the novel gene Claudin 13 (*Cldn13*), a member of the Claudin family of tight junction proteins using RNA expression, microarray and phylogenetic analysis. We present evidence that *Cldn13* appears to be co-ordinately regulated as part of a stress induced erythropoiesis pathway and is a mouse-specific gene mainly expressed in tissues associated with haematopoietic function. CLDN13 phylogenetically groups with its genomic neighbour CLDN4, a conserved tight junction protein with a putative role in epithelial to mesenchymal transition, suggesting a recent duplication event. Mechanisms of mammalian stress erythropoiesis are of importance in anaemic responses and expression microarray analyses demonstrate that *Cldn13* is the most abundant Claudin in spleen from mice infected with *Trypanosoma congolense*. In mice prone to anaemia (C57BL/6), its expression is reduced compared to strains which display a less severe anaemic response (A/J and BALB/c) and is differentially regulated in spleen during disease progression. Genes clustering with *Cldn13* on microarrays are key regulators of erythropoiesis (*Tal1*, *Trim10*, *E2f2*), erythrocyte membrane proteins (*Rhd* and *Gypa*), associated with red cell volume (*Tmcc2*) and indirectly associated with erythropoietic pathways (*Cdca8*, *Cdkn2d*, *Cenpk*). Relationships between genes appearing co-ordinately regulated with *Cldn13* post-infection suggest new insights into the molecular regulation and pathways involved in stress induced erythropoiesis and suggest a novel, previously unreported role for claudins in correct cell polarisation and protein partitioning prior to erythroblast enucleation.

## Introduction

The Claudins are a family of more than 23 small (20–27 kDa) tetraspan transmembrane proteins[1] which, alongside occludin, are the major components of tight junction (TJ) filaments in epithelial and endothelial cells. Tight junctions act as a primary barrier to the diffusion of solutes through the intercellular space and also have an important role in creating a boundary between the apical and the basolateral plasma membrane domains, allowing the specialized functions of each surface to be maintained [2]. As well as paracellular ion transport, TJs play a role in recruiting various cytoskeletal and signalling molecules at their cytoplasmic surface. TJ proteins therefore, play critical roles in cellular proliferation and neoplastic pathways by linking extracellular proteins to intracellular signalling pathways and the cytoskeleton [3], [4], [5]. Intracellularly, Claudins are connected with several TJ-associated proteins, including TJP1, 2 and 3 (ZO-1,2,3), INADL (PATJ) and MPDZ (MUPP1) [6], via a C-terminal PDZ-binding motif. MPDZ is an interacting partner of the receptor (KIT) for the haemopoietic cytokine stem cell factor, KITL (SCF) [7]. The activity of members of the Claudin family (Claudins 1, 2, 3, 4 and 7) is influenced by the transcription factors SNAI1 and 2 [8], [9], [10], which are key regulators of epithelial mesenchymal transition, and various kinases including protein kinase A (PKA) and protein kinase C (PKC) [11]. In the case of Claudins 1 and 2, their regulation by SNAI1 is downstream of TGFβ signalling mediated by the PI3K and MEK pathways [9].

Stress induced erythropoiesis is a process invoked under conditions of anaemia and requires significant proliferation of progenitor cells before terminal differentiation processes are invoked. In adult mammals the usual site of erythropoiesis is bone marrow, however, under conditions of anaemic stress (e.g. caused by acute bleeding or parasitic infection) the spleen can become a major site of red blood cell (RBC) production. This is observed as increased numbers of erythropoietic islands, the functional units of erythropoiesis, comprising a central macrophage surrounded by erythrocytic cells at various stages of maturation. In addition, in adult mice (but not humans) a significant proportion of extra-medullary erythropoiesis normally occurs in spleen [12]. The master regulator of erythropoietic activity is erythropoietin (EPO) which is transcriptionally controlled by hypoxia-inducible factor-1alpha (HIF1A) [13], [14]. Reduced tissue oxygen levels, as observed in anaemia, induce upregulation of EPO by HIF1A and a subsequent rise in RBC production. Under conditions of stress this system is modulated by other factors including bone morphogenetic protein-4 (BMP4) and KITL (SCF) which are essential for responsiveness of erythroid progenitors to EPO signalling [14]. Phosphatidylinositol 3-kinase (PI3K) enzymes regulate key signal transduction pathways controlling cell processes implicated in carcinogenesis and PI3K signalling downstream of both EPO and KITL, is thought to co-ordinate stress induced erythropoietic expansion [15], [16].

*Trypanosoma congolense* is a tsetse fly-transmitted intravascular protozoan parasite causing severe, acute or chronic disease (trypanosomosis) in mammals including cattle and other livestock and consequently affects development and economic growth in sub-Saharan Africa. In cattle, consistent features of trypanosomosis are anaemia and sporadic episodes of fever. Infected animals exhibit leukopenia, weight loss and enlargement of some organs (spleen and liver). Chronic infection is characterised by appetite loss, lethargy and emaciation and often death due to congestive heart failure[17]. In rats, infection with *T. congolense* initially causes an increase in medullary erythropoiesis and a reduction in the myeloid: erythroid cell ratio. Subsequently erythropoiesis declines, while granulopoiesis, megakaryopoiesis, plasma cell production and erythrophagocytosis increase [18].

It is hoped that understanding of trypanotolerance, as seen in some indigenous breeds of cattle, will advance disease control. Inbred strains of laboratory mice are used as a model system to study susceptibility to *T. congolense* infection: C57BL/6 mice are relatively tolerant but develop a severe anaemic response, whereas BALB/c and A/J strains, although regarded as susceptible (exhibit higher levels of parasitemia and early death), have relatively mild and transient anaemia [19]. Similarities between the anaemic responses of C57BL/6 mice and cattle to trypanosome infection have led to the use of this strain to identify pathways important for this feature of the disease [18], [19] and resulted in the hypothesis that reduced haematopoietic potential may be responsible for its relatively increased susceptibility to anaemia [20]. Importantly, the capacity to control anemia is thought to be the most significant factor contributing to trypanotolerance of cattle [21] and differences in erythropoietic potential have been suggested as contributing to susceptibility to the disease [22].

Here we report the characterization of a member of the Claudin family, Claudin 13 (*Cldn13*). Functionally, we have evidence from expression patterns and microarray data that *Cldn13* may play a role in a stress erythropoietic response to *T. congolense* induced anaemia. Our data implicate *Cldn13* and other previously unassociated genes in an erythropoietic pathway constitutively downregulated in mice prone to anaemia, thus providing potential new insights into the molecular regulation of erythropoiesis. It is probable that *Cldn13* is a mouse specific gene that arose as a result of a local gene duplication event.

## Results

### Claudin13 sequence structure and properties

**Figure 1:**
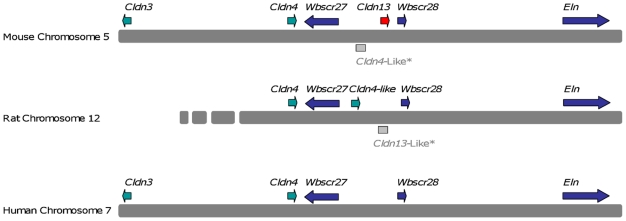
Comparison between transcript maps of mouse chromosome 5G2 containing *Cldn13* and syntenic regions of rat chromosome 12 and human chromosome 7q11.23 (part of the WBS region). Full length *Cldn13* (AF516681) is only present in mouse. The predicted rat ‘*Cldn4*-like’ transcript (XP_001066865) is only present in rat. *Rat *Cldn13*-like and mouse *Cldn4*-like are genomic sequences and there is no evidence that they are transcribed.

**Figure 2:**
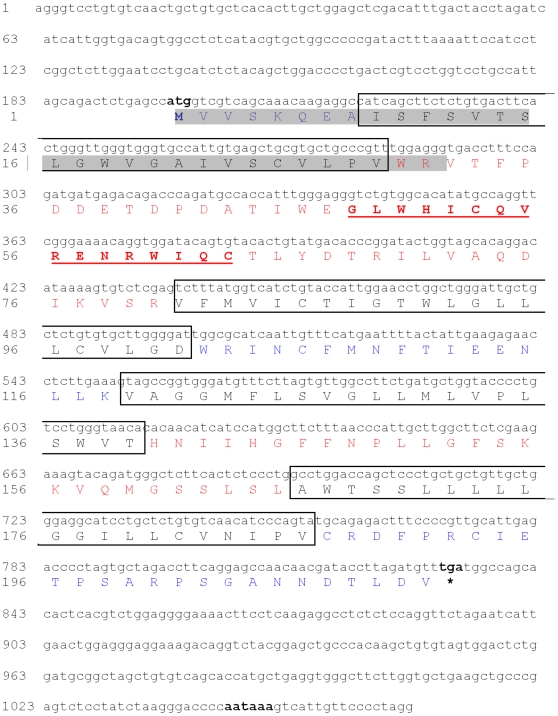
*Mus musculus Cldn13* sequence. *Cldn13* start (ATG, 198 bp), stop (TGA, 831 bp) codons, and poly-adenylation signal (aataaa, 1045 bp) are in bold. The four transmembrane regions are boxed (amino acid residues 9–21, 81–101, 119–139, 166–186). The Claudin signature (amino acid residues 48–63) is in bold and underlined and putative signal peptide (amino acid residues 1–31) is highlighted in grey. Predicted extracellular, transmembrane and intracellular domain residues are shown in red, black and blue respectively.

Our search for novel genes on mouse chromosome 5G2, syntenic to the Williams-Beuren Syndrome (WBS) region on human chromosome 7q11.23 [23], led to the identification of *Cldn13*. Screening an in-house mouse embryo cDNA library with segments from a BAC clone (gi: AC079938) identified a new gene, *Cldn13* (submitted to Genbank in 2002 gi: AF516681) with a PMP22_Claudin motif. *Cldn13* spans a genomic region of 1277 bp and is located 29579 bp centromeric to *Cldn4* (Fig. 1). The cDNA sequence is 1066 bp in length, and consists of two coding exons (825 bp and 241 bp; Table S1) encoding a protein of 211 amino acid residues with a predicted molecular weight of 23504 kDa and theoretical pI of 5.54 (Fig. 2). CLDN13 is predicted to contain four transmembrane domains, a conserved feature of the Claudin family (IPR004031, PF0082), and has two extracellular loops (residues 30–80 and 140–165). A conserved Claudin signature ([GN]-L-W-x(2)-C-x(7,9)-[STDENQH]-C, PDOC01045) is located between the first and second transmembrane regions (residues 48–63) (Fig. 2). Other predicted amino acid motifs are detailed in Table S2.

### *Cldn13* is a murinae specific gene

*Cldn13* sequences were not identified in any additional species, including humans, suggesting that *Cldn13* may be specific to *Mus musculus* and possibly other closely related rodents. In *Rattus norvegicus*, a 225 bp sequence with 78% sequence identity to the 3′ UTR of *Cldn13* was identified on chromosome 12 (AC091616). The overlapping genomic clone (AC091752) contains the rat *Cldn4* gene indicating that this is the region syntenic to the mouse chromosome 5 region containing *Cldn3*, *Cldn4* and *Cldn13*. There does not appear to be a full-length rat *Cldn13* ortholog since we failed to identify any sequences sharing significant identity to the first exon, suggesting that regardless of any earlier functional role, it has degenerated into a non-functional remnant in the modern rat (Fig. 1).

In addition, a predicted ‘*Cldn4*-like’ sequence was detected in rat (XP_001066865) which maps between *Cldn4* and the degenerate *Cldn13*-like sequence on rat chromosome 12 and shares sequence similarity with a mouse ‘*Cldn4*-like’ genomic sequence between *Cldns4* and *13* on mouse chromosome 5G2 (Accession number AC079938) (Fig. 1; Figs S1 and S2). There is no transcript evidence for these genomic sequences (mouse *Cldn4*-like and rat *Cldn13*-like) indicating that they are unlikely to be transcribed.

### *Cldn13* is expressed predominantly in tissues associated with haematopoiesis

**Figure 3:**
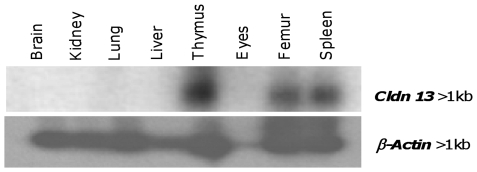
Expression of *Cldn13* mRNA in adult mouse tissues. Adult mouse tissue Northern blot showing the tissue specific distribution of *Cldn13* expression (upper panel) with β*-actin* as a loading control (lower panel).

Northern blot analysis with a *Cldn13* specific probe identified a single mRNA transcript (>1 kb) in adult mouse thymus, femur and spleen; no bands were observed in brain, kidney, lung or liver (Fig. 3). RT-PCR analysis confirmed *Cldn13* was present in adult mouse thymus, femur, spleen, ribs, skull, and muscle with barely detectable expression in brain and intestine. In newborns expression was detected in kidney, liver, lung, ribs, skull and spine (Table 1). During mouse development, *Cldn13* is expressed in embryos at stages 10.5, 11.5, 13.5 and 15 dpc but not at 9.5 or 17 dpc (Table 1). A more limited analysis of the expression patterns of *Cldn3* and *Cldn4* (which lie close to *Cldn13* on mouse chromosome 5) showed different profiles. Both were detected in adult spleen, bladder, intestine, lung, kidney and teeth, and in lung and skin of newborn mice. *Cldn3* is also expressed in adult liver, muscle and ribs and newborn liver and ribs and *Cldn4* in adult eyes and tongue (Table 1).

**Table 1:** RT-PCR expression analysis of Claudins on mouse chromosome 5G.

Tissue	*Cldn13*	*Cldn4*	*Cldn3*
***Adult***			
Bladder	−	+	+
Brain	+/−	−	−
Eyes	−	+	−
Femur	+	−	−
Kidney	−	+	+
Heart	−	−	−
Intestine	+/−	+	+
Liver	−	−	+
Lung	−	+	+
Muscle	+	−	+
Ribs	+	−	+/−
Skull	+	−	−
Spleen	+	+	+
Teeth	−	+	+
Testis	−	NT	NT
Thymus	+	−	−
Tongue	−	+	−
***Newborn***			
Brain	−	−	−
Eyes	−	−	−
Heart	−	−	−
Kidney	+	−	−
Liver	+	−	+
Lungs	+	+	+
Ribs	+	−	+/−
Skin	−	+	+
Skull	+	−	−
Spine	+	NT	NT
***Embryo***			
9.5 dpc	−	NT	NT
10.5 dpc	+	NT	NT
11.5 dpc	+	NT	NT
13.5 dpc	+	NT	NT
15 dpc	+	NT	NT
17 dpc (whole)	−	NT	NT

(+  =  strong expression; +/−  =  weak expression; −  =  not expressed; NT  =  not tested). All samples tested positive for the control gene, *β-actin* (data not shown).

The expression of *Cldn13* in haematopoietic tissues is further emphasised by publicly available data (BioGPS portal http://biogps.gnf.org/#goto=genereport&id=57255) from a global mouse gene expression analysis in 91 tissues including >20 haemopoietic cell lineages. Importantly, these data demonstrate exceptionally strong expression in mega-erythrocyte progenitor cells, bone marrow and bone [24].

### Phylogenetic analysis

**Figure 4:**
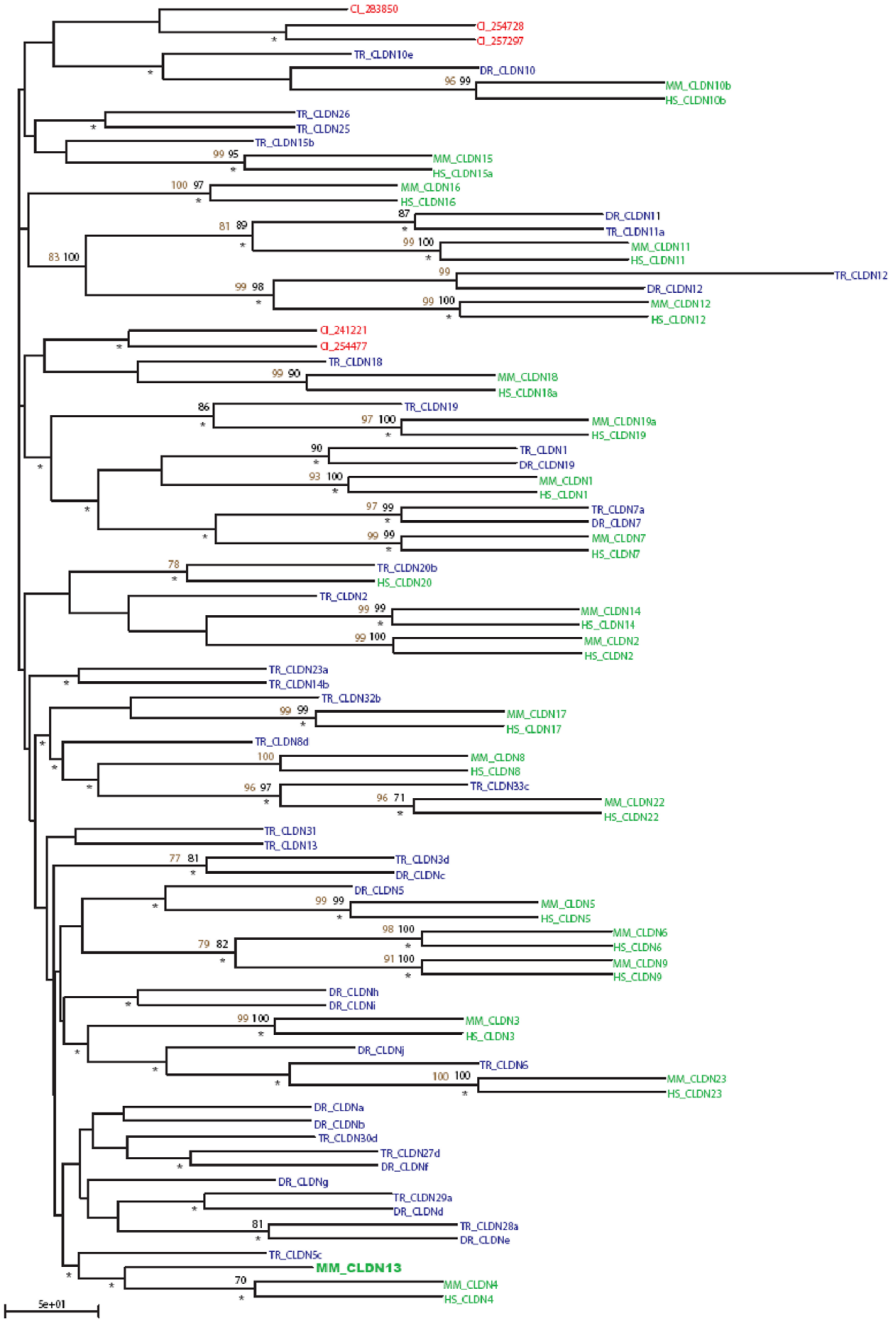
Neighbour Joining phylogenetic tree representing the Claudin protein family. The topology has been tested using bootstrap replicates (black numerals) and parsimony (brown numerals) (only scores >75% have been included). Agreement with maximum likelihood topology is indicated by an asterix (*). Species: *Ciona intestinalis* (red), teleost fish (*Tetraodon nigroviridis* and *Danio rerio*) (blue), and mammals (*Homo sapiens* and *Mus musculus*) (green). *Mus musculus* Claudin13 is highlighted in bold green font (full names and accession numbers of the abbreviations used are detailed in Table S5).

As *Cldn13* appears to be mouse specific, we explored its phylogenetic relationship with other Claudin family members. Eighty-nine sequences were collected (Table S3) and a robust phylogenetic tree generated using neighbor joining, bootstrapping, maximum likelihood and parsimonious methodologies (Fig. 4). Many regions of the consensus tree topology are well supported by all these methods. There is strong support for the pairings of CLDN 6 and 9, and CLDN 11 and 12, as well as for the groupings of CLDN 1, 7 and 19, CLDN 8, 17 and 22, and CLDN 3 and 23. The *Mus musculus* CLDN4 and CLDN13 clade is only strongly supported by maximum likelihood, however all statistical methods applied consistently reproduced this grouping, albeit with a reduced level of support (data not shown).

From this data we suggest that mammalian members of the Claudin protein family are orthologous, sharing a common ancestor prior to the human/mouse split. This is also true for many fish Claudins. *Danio rerio* is interesting because it appears to have followed two different routes during Claudin family development and, in addition to the orthologs it shares with other chordates, it possesses a group of paralogous alphabetically named Claudins [25]. This fish specific expansion was generated through gene duplication and development within the *Danio rerio* genome [25]. As expected, the *Ciona intestinalis* sequences behaved as outliers.

Of the 32 known members of the Claudin gene family only CLDN 5, 7, 10–12 and 19 appear to have orthologs in each of *H. sapiens*, *M. musculus*, *T. rubripes* and *D. rerio*. CLDN 25–33 are specific to *T. rubripes*. With the exception of CLDN13, mice and humans share the same complement of Claudins (Table 2). A *Takifugu rubripes* Claudin gene has been named *Cldn13* (AY554386) [25], however, this sequence fails to group with murine CLDN13 in our trees. It also displays minimal shared sequence similarity when compared to *Mus musculus* CLDN13 (Fig. S3) and reciprocal BLAST searches fail to identify a relationship between these sequences indicating that it is not a true ortholog.

**Table 2:** Species distribution of Claudins.

Claudin	*H. sapiens*	*M. musculus*	*D. rerio*	*T. rubripes*
1	*	*	--	*
2	*	*	--	*
3	*	*	--	*
4	*	*	--	--
5	*	*	*	*
6	*	*	--	*
7	*	*	*	*
8	*	*	--	*
9	*	*	--	--
10	*	*	*	*
11	*	*	*	*
12	*	*	*	*
**13**	--	*	--	* (#)
14	*	*	--	*
15	*	*	--	*
16	*	*	--	--
17	*	*	--	--
18	*	*	--	*
19	*	*	*	*
20	*	*	--	*
21	--	--	--	--
22	*	*	--	--
23	*	*	--	*
24	--	--	--	--
25	--	--	--	*
26	--	--	--	*
27	--	--	--	*
28	--	--	--	*
29	--	--	--	*
30	--	--	--	*
31	--	--	--	*
32	--	--	--	*
33	--	--	--	*

Distribution of members of the Claudin protein family across four species. Each row represents a single member of the Claudin family; (*) Claudin present; (--) Claudin absent. # *T.rubripes Cldn13* is unlikely to be a true homologue of the mouse gene (see results and Figure S3). Alphabetically named Claudins have been excluded as they are the result of a *Danio rerio* specific expansion [25].

### Claudin family expression in response to *T. congolense* infection in mice

**Figure 5:**
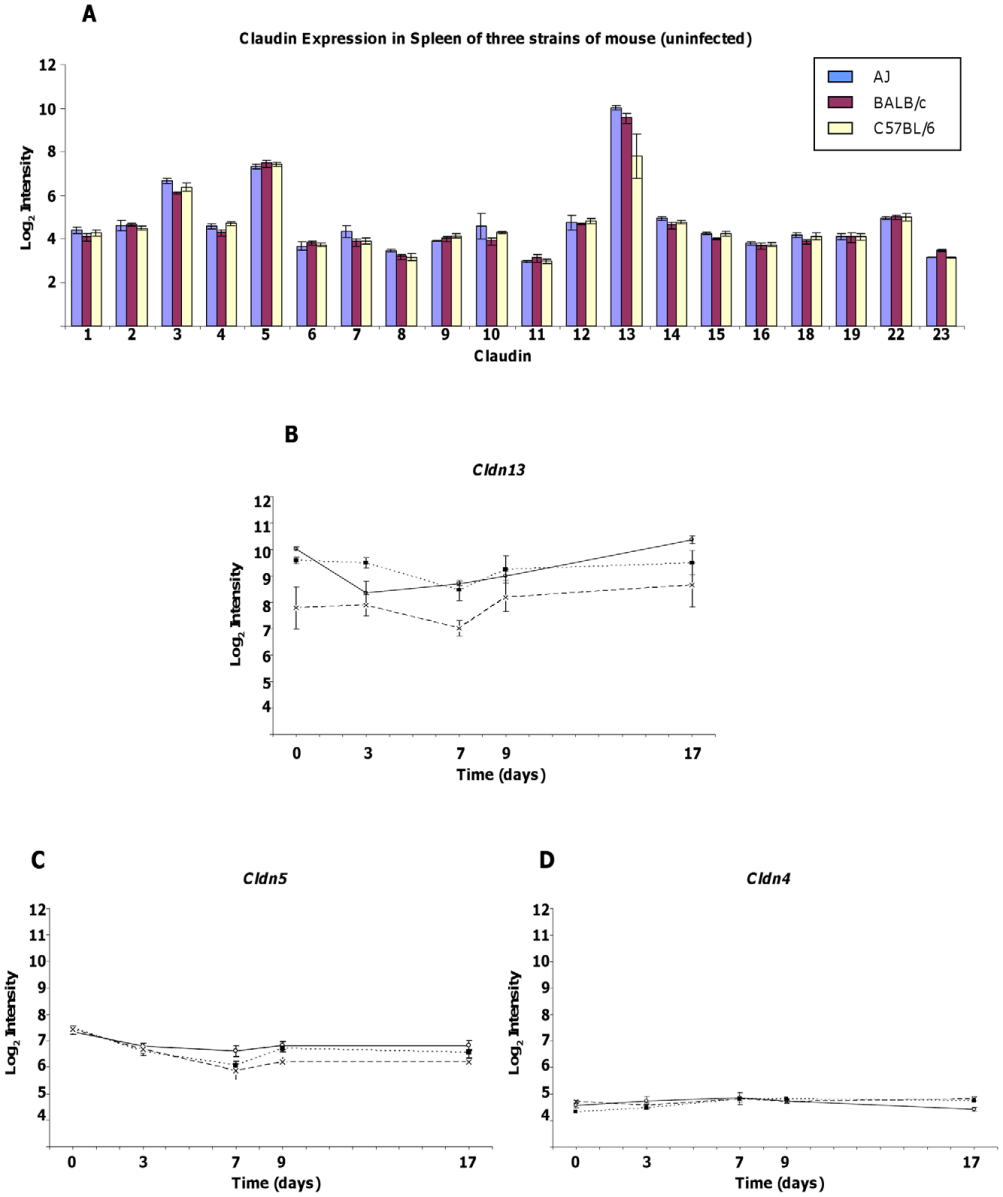
Expression of Claudin genes in mouse spleen in response to *T. congolense* infection. Data is expressed as mean Log_2_ intensity (±) standard error. **A**. Bar chart showing expression profiles of Claudin genes present on Affymetrix mouse microarrays prior to infection. *Cldn13* is the most abundant and levels of this transcript are depressed in C57BL/6 prior to infection. **B–D** Expression profile of *Cldn13*, *Cldn5* and *Cldn4* in microarray analysis over the course of *T. congolense* infection in three different strains (A/J – open circles, solid line; BALB/c – closed squares, dotted line; C57BL/6 – crosses, dashed line).

As *Cldn13* appears to be preferentially expressed in tissues associated with haematopoiesis/immunity (bone, spleen and thymus) we analysed microarray data derived from mice exposed to the pathogenic protozoan *T. congolense* to determine whether *Cldn13* levels were affected. In spleen, *Cldn13* is the most abundant of the twenty Claudins analysed. Log_2_ relative expression levels of *Cldn13* averaged across all three strains prior to infection are 9.12 (absolute value 770), compared to 7.41 (absolute value 170) of *Cldn5* (p = 0.0009), the second most abundant Claudin in spleen (Fig. 5A).

To explore early changes initiated in response to infection, *Cldn13 e*xpression levels were recorded in spleens of A/J, BALB/c and C57BL/6 (anaemia prone) mice, across five time points (days 0, 3, 7, 9, and 17 post infection) (Fig. 5B). Prior to infection *Cldn13* is 2.4–3.3 fold less abundant (p≤0.03) in C57BL/6 than in A/J and BALB/c (strains in which anaemia is transient). *Cldn13* levels remain significantly lower (2.8 fold; p≤0.02) at days 3 and 7 in C57BL/6 compared to BALB/c. C57BL/6 *Cldn13* expression is also reduced (1.4 and 3.0 fold at days 3 and 7, respectively) compared to AJ mice, however the difference only reaches significance at day 7 (p = 7.2×10^−5^). At later stages of infection (days 9 and 17), although expression in C57BL/6 still appears lower (1.2–1.9 fold), the strain differences do not reach significance (p≥0.08).

Of the other 19 Claudins, the largest strain specific differences observed were in *Cldn5*, which demonstrated 1.5–1.6 fold higher expression in AJ mice on day 7 (p = 0.04), and 1.5–1.6 fold higher expression in both AJ and BALB/c relative to C57BL/6 mice on day 9 (p = 0.01) (Fig. 5B). Levels of *Cldn4*, which is phylogenetically related to *Cldn1*3, were similar between all strains with marginal but significant differences only noted between BALB/c and C57BL/6 prior to infection, and between AJ and C57BL/6 on day 17 (1.3 fold higher in C57BL/6; p≤0.02, at both time points) (Fig. 5C). Differences in expression of other Claudins were marginal (Figs S4 and S5). Notably, only *Cldn13* demonstrated consistently reduced expression in the anaemia prone C57BL/6 mice relative to the other strains.

*Cldn13* expression also varied significantly post-infection over time (Fig. 5B). In A/J mice, *Cldn13* showed the largest, most significant changes in expression over the course of the experiment, with levels decreasing to 2.8 fold relative to those of uninfected mice at day 3 (p = 0.001) then increasing 3.6 fold between days 3 and 17 (p = 0.0009) to reach levels marginally higher (1.3 fold) than in uninfected mice at day 17. Expression in BALB/c and C57BL/6 mice reached a minimum (2.0 and 2.2 fold decreased respectively) at day 7 followed by recovery to pre-infection levels by day 17 in BALB/c and a substantial rise of *Cldn13* to above pre-infection levels (2.2 fold at day 17 versus day 0) in C57BL/6. However, this final increase was not significant (p = 0.19) and levels remained lower than those in AJ and BALB/c strains.

Of the other Claudins assayed, *Cldn5* also demonstrated significant changes in transcription levels over the course of infection in all three strains with initial decreases of between 1.6 and 2.7 fold (between days 0 and 7; p≤0.009). However, in contrast to *Cldn13*, *Cldn5* expression remained low (1.4 to 2.1 fold decreased at day 17 compared to day 0, p≤0.03) throughout the experiment (Fig. 5C). Changes in levels of the other Claudins did not follow a discernable pattern and were either minimal (≤1.5 fold change) or insignificant (p≥0.05) (Figs S4 and S5).

To summarise, in contrast to the expression of other Claudins, levels of *Cldn13* are lower in anaemia prone C57BL/6 mice compared to other strains prior to, and throughout infection. During the early stages of infection, expression of *Cldn13* decreases in all three strains, followed by subsequent increases to at or above levels seen in uninfected mice. These data suggest that *Cldn13* may be the major Claudin in mouse spleen and is likely to have functional significance in this tissue.

### Genes clustering with *Cldn13* in *T. congolense* infected mice share a common theme of erythropoiesis

To ascertain potential pathways in which *Cldn13* may participate, ten genes displaying expression patterns most similar to *Cldn13* (distance metric; computed using Pearson correlation [26]) were identified. Of these, nine were notable for their shared links to erythropoiesis (Table 3; Fig S6); three are key regulators of erythropoiesis (*Tal1*, *Trim10, E2f2*) [27], [28], [29], [30] and two are erythrocyte specific membrane proteins (*Gypa*, *Rhd*) [31], [32], [33]. *Tmcc2* is the homologue of human *TMMCC2* which has recently been strongly associated with mean corpuscular volume (MCV, red blood cell volume), a measure of anaemia, in a genome wide association study [34]. *Cdca8*, *Cenpk* and *Cdkn2d* have more indirect associations with erythropoietic pathways. *Cdca8* (*Borealin*) and *CenpK* (centromere protein K; *Solt1 -* Sox6 leucine zipper binding protein) are thought to be involved in mitotic chromosome segregation. *Cdca8* encodes a member of the centromeric chromosomal passenger complex (CPC) required for correct alignment and segregation of chromosomes [35]. The CPC consists of four proteins; CDCA8, BIRC5 (Survivin), INCENP and AURKB (Aurora kinase B). BIRC5 has recently been shown to be essential for proper haematopoietic development of erythro-myeloid precursors, being particularly crucial in erythroid differentiation [36]. In other systems, BIRC5 regulation of clonal expansion has been demonstrated to be downstream of PI3K and Akt signaling [37] and CDCA8 and BIRC5 undergo co-coordinated transcriptional repression in response to inactivation of CDK4 [38].

**Table 3:** Genes with expression profiles clustering most closely with *Cldn13* across two separate microarray datasets.

Gene	*Array	Accession Number	Name/Alias	Erythrocytic Function/Association
***Tal1***	1	NM_011527	T-cell acute lymphocytic leukaemia 1, Scl	Transcription factor necessary for erythropoiesis [30]
***Trim10***	1,2	NM_011280	Tripartite motif protein 10, Heff1	Regulator of erythropoiesis [28]
***E2f2***	1	BG967674	E2F transcription factor 2	Forms a complex with GATA1 and retinoblastoma protein (RB1), encourages terminal erythroid differentiation [29]
***Gypa***	1,2	M26385	Glycophorin A	Erythrocytic membrane protein; downstream of TAL1 [31]
***Rhd***	1,2	AF069311	Rh blood group, D antigen	Erythrocytic membrane protein [90]
***Tmcc2***	1	AK004359	Transmembrane and coiled-coil domains 2	Human *TMCC2* has been associated (GWAS) with red cell volume[34]
***Cdkn2d***	1	BC013898	Cyclin-dependent kinase inhibitor 2D, INK4D, p19Ink4d	Downstream target of KLF2, a transcription factor regulating erythropoiesis [91]
***CenpK***	1	NM_021790	Centromere protein K, Solt	Associated with SOX6, a key transcriptional regulator of erythropoiesis [41]
***Cdca8***	1	AV307110	Cell division cycle associated 8, Borealin	Associated with BIRC5 (Survivin), important for erythropoiesis [36]
***Cdr2***	1	NM_007672	Cerebellar degeneration-related 2, Yo	None reported.
***Tspo2***	2	NM_027292	Translocator protein 2	Involved in cholesterol distribution during erythropoiesis [92]
***Rhag***	2	NP_035399	Rhesus blood group-associated A glycoprotein	Erythrocytic membrane protein with a role in ammonia and methylammonia transport [93]
***Icam4***	2	NM_023892	Intercellular adhesion molecule 4	Glycoprotein expressed on red blood cells and erythroid precursor cells [94]
**Unknown**	2	AK020489	Unknown	None reported
***Kel***	2	NM_032540	Kell blood group	Erythrocyte glycoprotein [95]
***Hemgn***	2		Hemogen	Promotes myeloid progenitor cells expansion downstream of HoxB4 [96]
***Add2***	2	NM_013458	Adducin 2 (beta)	Actin-binding protein of the erythrocyte junctional complex involved in maintenance of erythrocyte shape and membrane stability [97]

* Array1: from spleens of mice at different stages of trypanosome infection;*Array 2: from 91 different mouse tissues/cell lines including various haemopoietic cell lineages [24], [46].

*CenpK* has been proposed as the homolog of the *Schizosaccharomyces pombe* protein Sim4 and, in common with the CPC, is thought to localize to centromeric kinetochores and be involved in chromosome segregation [39]. CENPK was originally identified as physically associated with the transcription factor SOX6 [40] which has a critical role in erythropoiesis [41], [42]. *Cdkn2d* (cyclin-dependent kinase inhibitor 2D; *p19Ink4d*) is a member of the Ink4, ‘Inhibitor of cyclin dependant kinase (CDK) 4’ family of repressors which prevent inappropriate progression into the cell cycle [43]. There is evidence that CDKN2D contributes to differentiation of a model system of transformed erythroid precursors, murine erythroleukaemia (MEL) cells, by inhibition of CDK6 [44].

All nine genes act in pathways known to be downstream of either EPO, BMP4 or KITL, suggesting that a possible role in the stress response to anaemia induced by *T. congolense* infection. The remaining clustered gene is *Cdr2* (cerebellar degeneration-related 2 or Yo), a protein which has recently been shown to be important for mitotic spindle formation and proliferation of dividing cells [45] providing a putative link with *CenpK* and *Cdca8*.

To provide external validation for this cluster analysis, genes with similar expression patterns to *Cldn13* were identified in a separate publicly available array experiment [46]. The ten genes most closely correlated (co-efficient >0.98), included three red blood cell associated genes (*Trim10*, *Gypa* and *Rhd*), also identified in our *T. congolense* arrays, and a further six with known associations to either erythrocyte-specific processes (*Tspo2*, *Rhag*, *Icam4*, *Kel*, *Add2*) or myeloid progenitor cell proliferation (*Hemgn*) (see Table 3. and references therein). The remaining clustered transcript (Accession No: AK020489) has no known function and little homology to known genes.

### Differences in Haemoglobin levels between mouse strains are consistent with a reduced erythropoietic response to infection in C57BL/6

**Figure 6:**
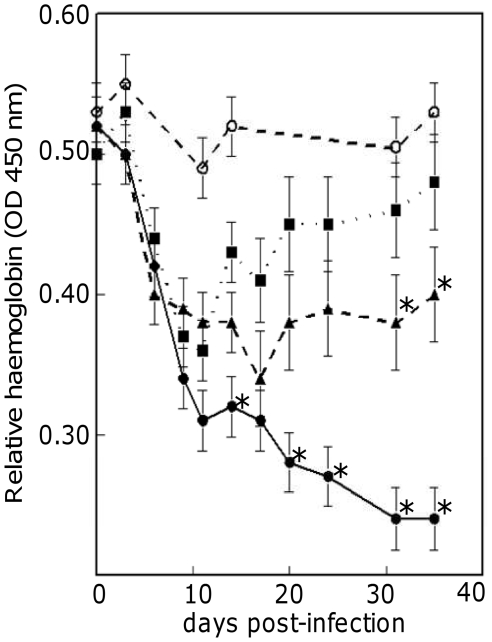
Haemoglobin levels in mouse strains during *T. congolense* infection. Relative haemoglobin titres, measured as OD at 540 nm with standard errors, in C57BL/6 (circles, full line), A/J (triangles, broken line) and BALB/c (squares, dotted line) after infection with *T. congolense*, and uninfected C57BL/6 mice (open circles, broken line). Each point is an average of triplicate samples taken from ten mice. The C57BL/6 values that were significantly lower than the A/J and BALB/c values are indicated by an asterisk (difference in value larger than twice the Standard Error of Difference). The last two haemoglobin values for A/J mice (indicated by asterisk) were significantly lower than the BALB/c values.

Levels of haemoglobin were assayed in blood samples from mice before and during infection with *T. congolense*. Relative concentrations of haemoglobin during the course of infection fell steeply in all three mouse strains in the initial stages of trypanosome infection. However, after day 9 and day 17 respectively, haemoglobin concentrations began to recover in BALB/c and A/J strains, whereas in C57BL/6 mice they continued to decline throughout the experiment (Fig. 6). Haemoglobin titres were lower in C57BL/6 from day 7 post infection and thereafter. The difference was significant on days 13 and 20–35 (p<0.001) (Fig. 6). AJ mice demonstrate haemoglobin levels intermediate to those observed in Balb/c and C57BL/6 and had significantly lower titres than Balb/c at the final two timepoints.

These results, alongside the reduced transcription of *Cldn13* and clustered erythropoietic gene, are all consistent with a reduced stress erythropoietic response in C57BL/6 in comparison with the other inbred strains. Levels of *Cldn13* and clustered genes associated with erythropoiesis also demonstrate a parallel decrease alongside haemoglobin levels in the initial stages of infection in all strains and begin to recover, suggesting nascent splenic erythropoiesis, prior to the recovery of haemoglobin levels in AJ and BALB/c. The failure of haemoglobin levels to recover in C57BL/6 and the subsequent chronic anaemia observed [20] may be reflected in the inflated expression of erythropoietic genes at later stages of infection as the organism attempts, unsuccessfully, to restore sufficient RBC capacity.

## Discussion

Phylogenetic profiling is useful for inferring functional and evolutionary relationships and previous work has positioned *Cldn13* in a clade with *Cldn2*, *14* and *20* [25], paired it with *Cldn23* (ORF5, BAB26222) [47], or failed to place it at all due to its inconsistent positioning within the tree [48]. In contrast, our phylogenetic data, generated from the consensus of four methods, highlights a relationship between *Mus musculus Cldn13* and *Cldn4/CLDN4*. *Cldn4* has been implicated in a variety of processes as diverse as osmotic response in the kidney [49] and blastocyst formation [50] and *CLDN4* has been shown to be both up and downregulated in various malignancies (reviewed in [51]). Mouse *Cldn4* is downregulated in EMT in response to SNAI1 [10] and expression of CLDN4 decreases the potential of pancreatic cancer cells to metastasize downstream of both MEK and PI3K [52]. Claudins evolutionarily associated with human *CLDN3* and *4* on chromosome 7 appear to have undergone duplication. The presence of a predicted ‘*Cldn4*-like’ sequence in rat (between *Cldn4* and the degenerative *Cldn13*-like sequence on rat chromosome 12) but with no transcript evidence, suggests a gene duplication prior to mouse/rat speciation events.

In support of our hypothesis that *Cldn13* is involved in erythropoiesis, a recent report identified it as one of a number of genes whose expression is significantly higher in erythroid cells [53]. Further evidence comes from microarray analysis of a *D. rerio* mutant model of haematopoiesis, *cloche*, which identified *Cldng* (topologically groups with *M. musculus* CLDN13/CLDN4 in our analysis) as differentially expressed between mutant and wild type zebrafish and predominantly found in erythroid cell lineages [54].

Haemopoietic cells are not generally thought to connect via intercellular junctions such as tight junctions although in a rare form of human acquired dyserythropoiesis, erythroblastic synartesis, septate-like membrane junctions (invertebrate septate junctions are thought to be analogous to TJs) and invaginations between erythroblasts which are tightly linked together have been observed [55]. This abnormality is responsible for ineffective erythropoiesis in patients leading to severe anaemia with reticulocytopenia. Cell-cell contacts do, however, occur within erythroblastic islands (erythrocyte-erythrocyte and erythrocyte-macrophage) and are important for successful erythropoiesis [56]. Erythrocyte-macrophage protein (EMP), intercellular adhesion molecule-4 (ICAM4, highlighted by our cluster analysis) and integrins α_4_β_1_ and α_v_ participate in these interactions and it is possible that CLDN13 may also contribute in some way.

The erythrocyte specific membrane proteins GYPA and RHD, also encoded by genes clustering with *Cldn13*, form part of a macromolecular erythrocyte membrane structure incorporating the Band-3 complex [57]. Given the known association of TJ complex components (albeit in non-erythroid cells) with the erythrocyte cytoskeletal component protein EPB4.1 (4.1R) [4], a protein involved in erythropoietic mitosis through interaction with the Band-3 complex [58], [59], it is possible that CLDN13 and other TJ components also function in this system.

**Figure 7:**
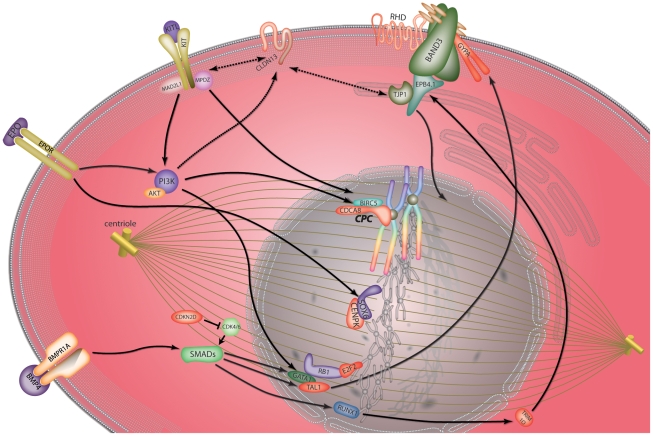
Predicted model of co-regulated gene behaviour in response to *T. congolense* mediated anaemic stress. Diagram represents an erythroblast cell during mitotic replication under conditions of stress. Proteins encoded by genes clustering with *Cldn13* in microarray data are in red. KITL and its receptor KIT signal through PI3K [15] and regulate BIRC5 (Survivin) and CDCA8 (Borealin) expression through interaction with MAD2L1 (MAD2) [36], [84], [85]. PI3K/AKt phosphorylate and activate GATA1 [86] which physically interacts with retinoblastoma (RB1) and E2F2 [29]. PI3K has been shown to be upstream of Claudin family members [9], potentially linking this pathway with CLDN13 (broken arrow). Claudin family members have been shown to physically interact with MPDZ (MUPP1) [87] (a binding partner of KIT) and TJP1 (ZO-1), a TJ component which interacts with EPB4.1 (4.1R) [4] (a key cytoskeletal component linking mitotic microtubules to the plasma membrane Band-3 complex [59]). Under hypoxic conditions BMP4 signals through SMAD proteins (including SMAD5) [62], [63], [88] a cascade regulating the transcription factors TAL1, GATA1, ELKF (not shown) and RUNX1 [62], [88]. Downstream targets of these factors include TRIM10 (HERF1) [28], RHD and GYPA [31], [89]. Progression through the cell cycle may be controlled by regulation of CDK4/6 by CDKN2D [43]. Artwork by www.wishpress.com.

The role of EPB4.1 in the regulation of mitotic chromosome segregation in erythroid cells [60] also suggests a link between this protein and *Cdca8*, *Cenpk* and *Cdr2* clustered with *Cldn13* in our microarray data. The clustered gene *Trim10* is an important regulator of erythropoiesis downstream of the key transcriptional regulator of haematopoiesis RUNX1 (AML1, core binding factorβ) [28] and determines production of an alternatively spliced isoform of EPB4.1 which is indicative of erythrocytic maturation [61]. Recent work suggests integration of the RUNX1/TAL1 haematopoietic pathways via the Smad family of transcriptional regulators downstream of BMP4 [62] which, in conjunction with KITL, is a pivotal mediator of hypoxia-induced stress erythropoiesis in mouse spleen [14], [63]. Another of the genes clustered with *Cldn13*, *E2f2*, has been shown to form a complex with RB1, a key player in the regulation of the G1-to-S-phase transition of the cell cycle [64] and GATA1, encouraging terminal erythroid differentiation by shutting down cell proliferation [29]. Although several of the clustered genes are established erythrocyte-associated genes (*Tal1*, *Trim10*, *E2f2*, *Gypa*, *Rhd*) the involvement of others (*Cdkn2d*, *Cdca8*, *Cenpk*, *Cdr2*, *Cldn13*) has not been proven. However, our data, along with evidence of association between *Cdkn2d*, *Cdca8* and *Cenpk* with factors essential in erythrocyte maturation strongly suggest a role for these genes in this process. Figure 7 provides a hypothetical view of how the genes identified through cluster analysis with *Cldn13* in *T. congolense* infected mice, may interact in developing erythroid cells.

One of the final steps in red blood cell maturation is enucleation. Chromatin becomes condensed and the nucleus migrates to one pole of the erythroblast before separating surrounded by plasma membrane and a small rim of cytoplasm [65], [66]. Thus the process could be regarded as an asymmetric form of mitosis. Many membrane proteins, including GYPA are preferentially located in the membrane of the mature reticulocyte rather than the extruded nucleus [67]. Given the role of Claudins in defining domains within the plasma membrane as part of TJs, and the involvement of the proteins we have identified as co-regulated during stress erythropoiesis in cell cycle checkpoint regulation and/or mitotic chromosome segregation, we suggest a potential new role for Claudins in correct cell polarisation and protein partitioning prior to erythroblast enucleation. However, further functional work is required to test this hypothesis.

The pattern of *Cldn13* regulation observed over the course of *T. congolense* infection is consistent with a stress induced erythropoietic response to parasite induced anaemia. We speculate that the downregulation of CLDN13 at the early stages of the stress erythropoietic response may influence the transition to a proliferating phenotype among splenic RBC pre-cursors. It is possible that this is mediated through reduced contacts with supportive stromal cells undergoing EMT as observed in fetal liver [68]. The recovery of *Cldn13* levels in later stages of infection may indicate populations of erythrocytic cells undergoing terminal differentiation, including enucleation, preceding the recovery from anaemia observed in strains A/J and BALB/c. The fact that expression of *Cldn13* and clustered genes are constitutively lower in C57BL/6 mice than in A/J or BALB/c mice is consistent with them representing a snapshot of an erythropoietic pathway permanently depressed in this anaemia prone strain. Further, levels of expression of these genes in C57BL/6 mice fail to reach those seen in A/J or BALB/c mice, consistent with the previous suggestion of a suppressed stress erythropoietic response contributing to the chronic anaemia observed in this strain [20].

Given the expression pattern of *Cldn13* in most haemopoietically active tissues (thymus, bone marrow and fetal liver) we do not exclude the possibility that it may also function in the generation of other blood cell lineages. Indeed, Claudin positive cells have been observed at the margins of distinct structures within the medulla of the thymus known as Hassall's corpuscles (HCs)[69] which are thought to function in production of immunosuppressive regulatory T cells. It remains unclear whether thymic *Cldn* expressing cells have functional TJs, although these structures were not detected between *Cldn* expressing cells in mouse [70].

In agreement with our RT-PCR expression data, expression of *Cldn13* has previously been reported in mouse intestine [71], [72] and in neonatal but not adult kidney [73]. There has also been a report of *Cldn13* expression in mouse bladder [74], however, we did not find evidence for this in our experiments, which may be due to differences in assay sensitivity as publicly available micro-array data [24] demonstrate minimal expression in this organ. It is also interesting to note decreased expression of *Cldn5* during *T. congolense* infection in all three inbred strains. Rat CLDN5 has been observed as part of atypical TJs within the splenic sinus endothelium, a structure associated with removal of senescent RBCs [75]. Given the almost complete identity of the protein sequence of mouse CLDN5 with that of rat (only two amino acids of 218 differ) we speculate that mouse CLDN5 may also function in these structures. Reduction in levels of *Cldn5* transcript in the spleens of all three inbred strains may be demonstrative of disrupted splenic architecture in the face of the massive haemophagocytosis in this organ in response to infection and/or the transformation of the spleen to erythropoietic function.

In conclusion, our data provides evidence of a role for *Cldn13*, and three other genes with no previous link to erythropoietic function (*Cdca8*, *Cenpk* and *Cdr2*), in a known stress response pathway with specific relevance in resistance to trypanosome infection and potential importance in many other contexts including acute anaemia and erythroleukaemia. Protein sequence analyses indicate that *Cldn13* is a member of the Claudin family and as such may well contribute to TJ formation. However, *Cldn 13* is an unusual gene; its distribution is apparently limited to the murinae, and belongs to a clade encompassing recently duplicating genes evolutionarily linked to human *CLDN3* and *CLDN4*. The most parsimonious explanation for the absence of *CLDN13* in humans, and all other species surveyed, is a gene duplication event specific to the murinae which lead to the evolution of a functional *Cldn13* gene in *Mus musculus*, but appears to have been lost in *Rattus norvegicus* leaving only the degenerate sequence we identified. Since *Cldn13* appears to be mouse specific, we speculate that other members of the Claudin family (possibly *CLDN4*) may be involved in erythropoiesis in other mammals and, given the widespread use of animal models to predict biological functions in humans, it will be important to clarify if this is the case.

## Methods

### Ethics Statement

Animals were housed at the International Livestock Research Institute (ILRI), Nairobi, Kenya. Mice used were 5–8 weeks of age. Animals received food and water ad libitum. All experimental procedures were approved by the Institutional Animal Care and Use Committee (IACUC, Licence No. 2004.7) at ILRI.

### RNA isolation

RNA was isolated from mouse tissues for northern blots and RT-PCR by extraction with total RNA isolation reagent (ABgene) according to manufacturer's instructions. mRNA was purified from total RNA using the Nucleotrap kit (ABgene) according to manufacturer's instructions. For microarray analysis, RNA was prepared from mouse liver and spleen using Trizol Reagent (Invitrogen) as described previously [76].

### PCR and RT-PCR

cDNA was prepared from 2 µg total RNA (pre-treated with DNaseI) using the ReverseIT 1^st^ strand synthesis kit (ABgene). PCR was carried out using the ReddyMix PCR system (ABgene) according to manufacturer's instructions. PCR primers are detailed in Table S4.

### Mouse Tissue Northern Blots

mRNA from each tissue (1 µg) was added to 4 µl RNA loading buffer without ethidium bromide (Sigma) and heated to 65°C for 10 min before loading on a 1% MOPS Formaldehyde gel (electrophoresis for ∼3 h at 5–6 mA/cm). 3 µl RNA markers (Sigma), in 2 µl ethidium bromide (75 µg/ml), were used on each gel. Gels were blotted overnight in 20× SSPE. Hybridization was in ‘Rapid-Hyb’ (Clontech) using P^32^ labelled PCR probes (Random Primers Labelling System GibcoBRL). The filters were washed at 60°C (0.2XSSC+0.1%SDS) and exposed to X-ray film at −70°C. *Cldn13* probe was generated by PCR amplification of a 588 bp fragment from mouse genomic DNA (Table S4); this region is outside the Claudin domain to avoid cross-hybridisation with other Claudin genes.

### Sequence collection and Phylogenetic analysis

A total of 121 *Homo sapiens*, *Mus musculus*, *Danio rerio* and *Takifugu rubripes* full length Claudin sequences were retrieved from GenBank (http://www.ncbi.nlm.nih.gov). This sequence set was supplemented with *Ciona intestinalis* sequence (obtained by searching with sequence ci134049, (http://genome.jgi-psf.org/Cioin2/Cioin2.home.html)). To simplify the phylogenetic analysis, a single representation of each entity was used and only the longest isoforms selected. This yielded a dataset totalling 89 sequences (Table S3). Claudin amino acid sequences were aligned using ClustalX [77] (http://bips.u-strasbg.fr/fr/Documentation/ClustalX/). Gap-containing sites were removed from the alignment and Maximum Likelihood trees were inferred using ProML from the PHYLIP package (http://evolution.genetics.washington.edu/phylip.html) [78]. The JTT model of amino acid substitutions was used with global rearrangement and correction for rate heterogeneity (α obtained from TREE-PUZZLE (http://www.tree-puzzle.de/) [79]. Neighbour joining bootstrap replicates and Bayesian inference methods were used to test tree topology using PHYLIP (Neighbour, ProtDist, SeqBoot) and Mr Bayes (http://mrbayes.csit.fsu.edu/) [80] respectively. PHYLIP was also used to determine the parsimonious topology (seqBoot, protparse, consense).

### Mouse model of African trypanosomiasis

*T. congolense* infection of mice is an established and valuable model of anaemia and is appropriate to use for investigating the association between gene expression and anaemia [17], [20]. Clone IL 1180 [81] was grown in sub-lethally irradiated Sprague–Dawley rats, and trypanosomes were isolated from infected rat blood by anion exchange column [82]. Mice were infected by intraperitoneal injection (1×10^4^ parasites in 200 µl of phosphate-buffered saline (pH 8.0) containing 1.5% glucose).

### Microarray hybridization and cluster analysis

Affymetrix Mouse 430 2.0 microarrays (http://www.affymetrix.com) were used to determine expression of genes in each of A/J, BALB/c and C57BL/6 mice. For each strain RNA was prepared from spleens of thirty mice. The samples were then mixed into five pools of five samples for each strain. This pooling strategy is predicted to give the same power to detect differential expression as fifteen individual samples hybridised separately [83]. Chips were initially assessed using DChip criteria to screen for outlier and normalised by RMA. The data are available through ArrayExpress (Accession No; E-MEXP-1190). P values for differences in gene expression (by time or strain) were calculated using the Student's T-test. Genes with expression patterns most closely matching those of *Cldn13* during infection were identified using a distance metric (Maxd software suite [26]).

### Haemoglobin Measurement

Samples of 2 µl of blood were collected from the tail and diluted in 150 µl of distilled water in 96 well plates. After 30 minutes at room temperature, the plate was centrifuged (600 x g, 10 min), 100 µl of supernatant transferred to a new plate and the relative haemoglobin concentrations measured by spectrophotometrical determination of optical density at 540 nm in an ELISA plate reader (Multiscan MCC/340, Titertek Instruments, Huntsville, AL, USA). Measurements were carried out in triplicate. The data were analysed by ANOVA (analysis of variance) using strain, time and their interaction. Subsequently mice were euthanised to allow tissue collection for microarrays, therefore observed changes in haemoglobin levels were not due to repeated sampling. All mice were euthanised after day 35 of the experiment.

## Supporting Information

Figure S1 ClustalW alignment of rat predicted CLDN4-like protein sequence with rat CLDN4, mouse CLDN4 and 13, and human CLDN4. Rat sequence XP_001066865 is unlikely to be an ortholog of mouse Claudin 13 as it shares more sequence similarity with mouse, rat and human Claudin 4.
(0.71 MB TIF)

Figure S2 tBLASTx alignments using Rat Cldn4-like protein sequence as the query. A genomic sequence with a high degree of similarity to rat ‘Cldn4-like’ is located on mouse chromosome 5 between Wbscr27 and Cldn13.
(0.28 MB TIF)

Figure S3 Putative Fugu Claudin 13. A. ClustalX alignments highlighting the relatively weak alignment of between mouse Claudin 13 (AY554386) and Fugu Claudin 13 (AF516681). B. Contrasting strong alignments between mouse Claudin 3 (NP_034032) and Fugu Claudin 3 (AAT64047). Combined with phylogenetic evidence this indicates that the suggested Fugu Claudin 13 is unlikely to be the true ortholog of mouse Claudin 13.
(0.53 MB TIF)

Figure S4 Data is expressed as mean Log2 intensity (± standard error. Expression profiles of Claudins 1–3 and 6–11 in microarray analysis over the course of *T. congolense* infection in three different strains (A/J - open circles, solid line; BALB/c - closed squares, dotted line; C57BL/6 - crosses, dashed line).
(0.34 MB TIF)

Figure S5 Data is expressed as mean Log2 intensity (± standard error. Expression profiles of Claudins 12, 14–16, 18, 19, 22 and 23 in microarray analysis over the course of *T. congolense* infection in three different strains (A/J - open circles, solid line; BALB/c - closed squares, dotted line; C57BL/6 - crosses, dashed line).
(0.22 MB TIF)

Figure S6 Data is expressed as mean Log2 intensity (±) standard error. A–I: Expression profiles of nine of the ten genes with expression patterns correlating most closely to those of *Cldn13* in microarray analysis of *T. congolense* infection in three different strains (A/J - open circles, solid line; BALB/c - closed squares, dotted line; C57BL/6 - crosses, dashed line). All of these are either known to function in erythropoiesis or have more indirect associations with erythropoietic pathways.
(0.43 MB TIF)

Table S1 (0.03 MB DOC)

Table S2 (0.03 MB DOC)

Table S3 (0.12 MB DOC)

Table S4 (0.03 MB DOC)
