# Supplementary figures and images for: Claudin 13, a Member of the Claudin Family Regulated in Mouse Stress Induced Erythropoiesis
Source: PLoS One. 2010 Sep 10;5(9):e12667. doi: 10.1371/journal.pone.0012667 (PMC2937028; doi:10.1371/journal.pone.0012667)

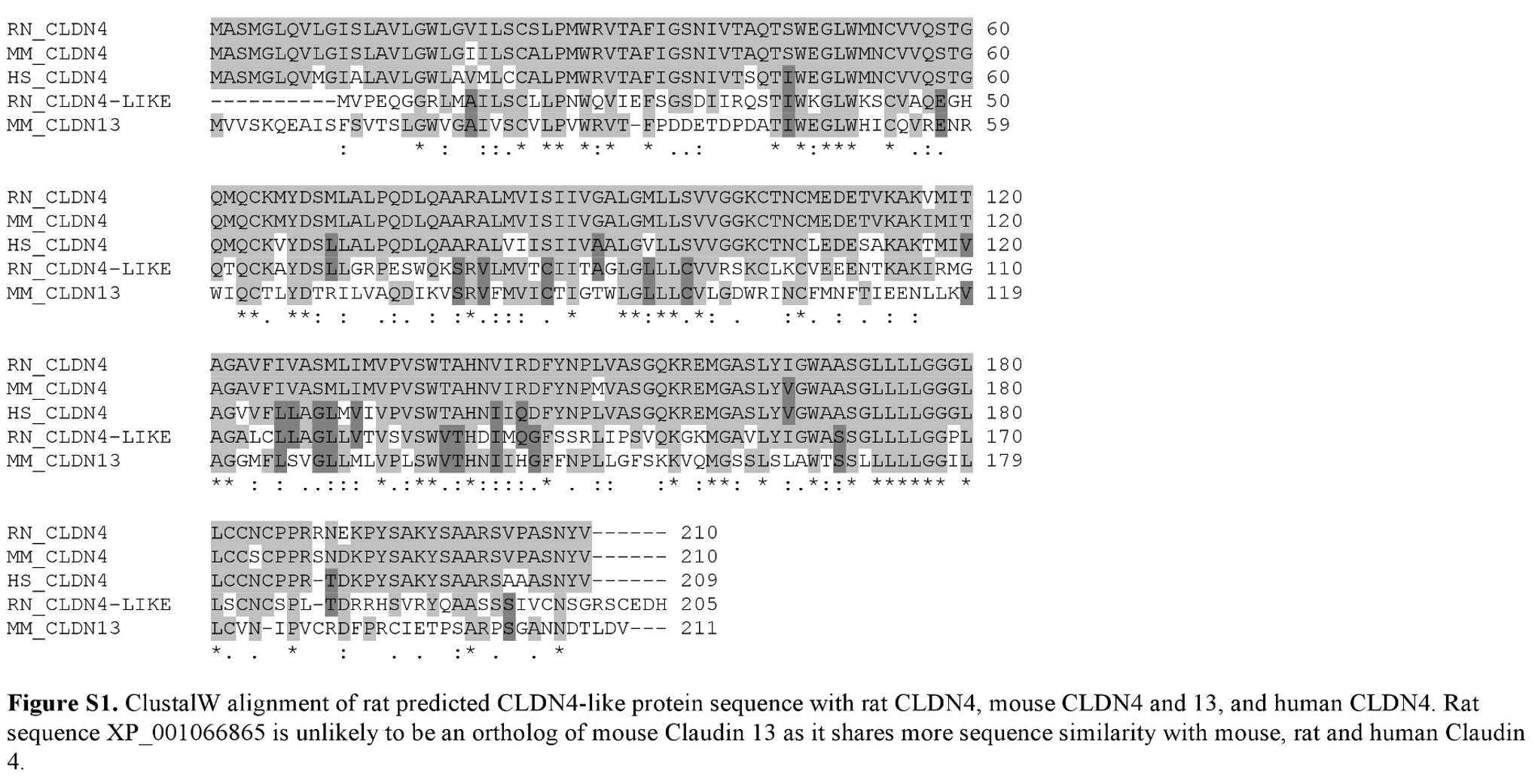

Supplement: Figure S1 — ClustalW alignment of rat predicted CLDN4-like protein sequence with rat CLDN4, mouse CLDN4 and 13, and human CLDN4. Rat sequence XP_001066865 is unlikely to be an ortholog of mouse Claudin 13 as it shares more sequence similarity with mouse, rat and human Claudin 4. (0.71 MB TIF) [file pone.0012667.s001.tif]

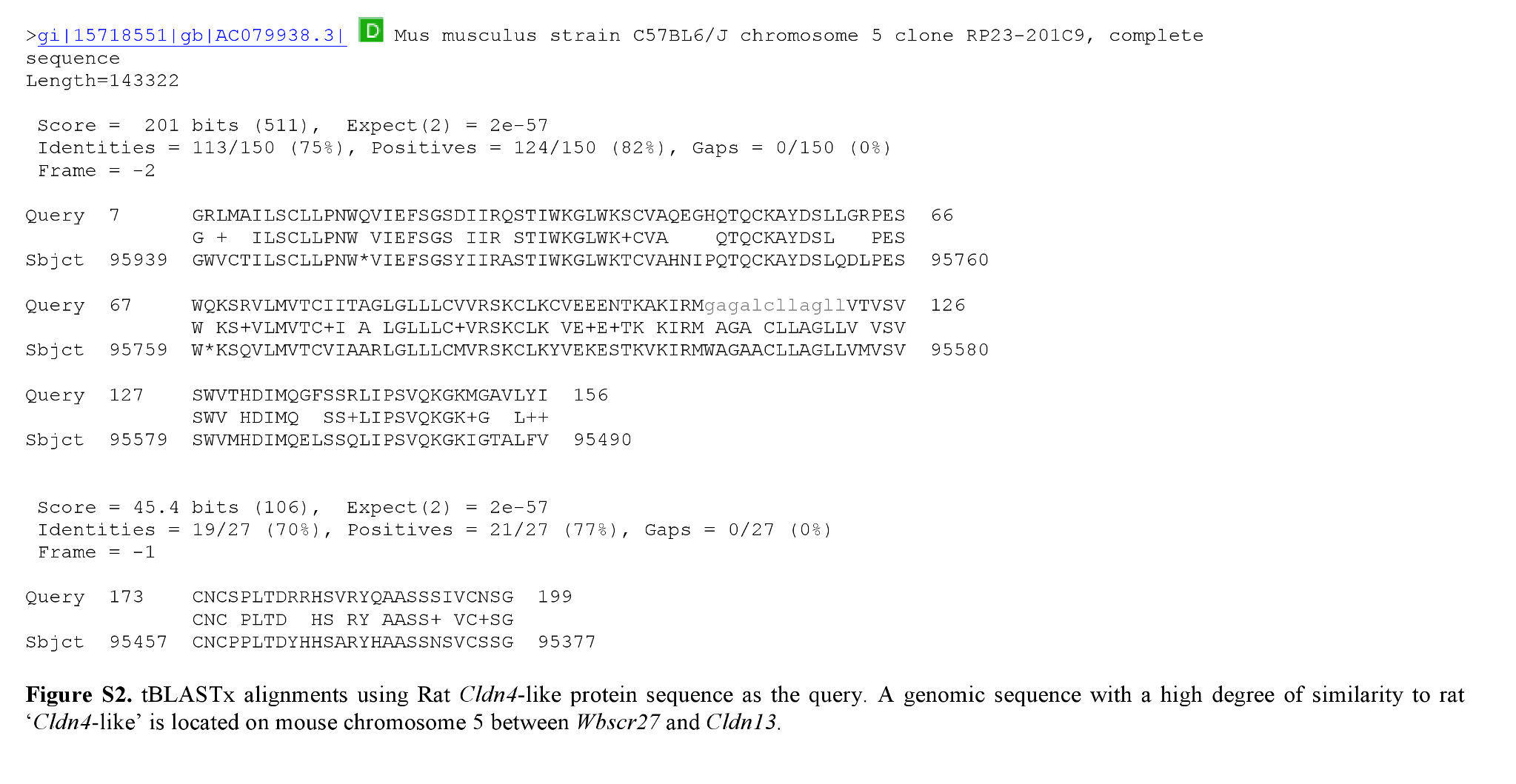

Supplement: Figure S2 — tBLASTx alignments using Rat Cldn4-like protein sequence as the query. A genomic sequence with a high degree of similarity to rat ‘Cldn4-like’ is located on mouse chromosome 5 between Wbscr27 and Cldn13. (0.28 MB TIF) [file pone.0012667.s002.tif]

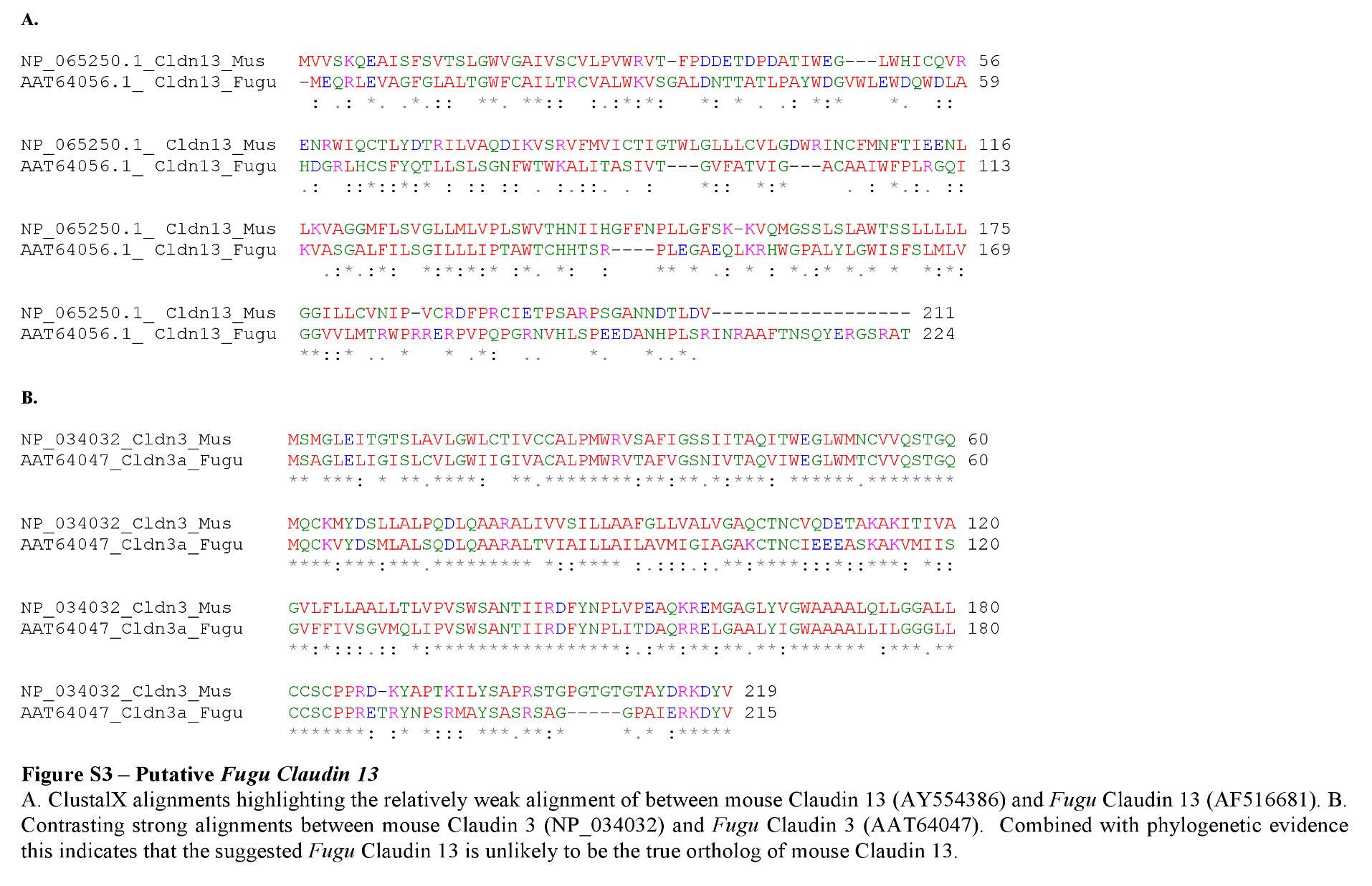

Supplement: Figure S3 — Putative Fugu Claudin 13. A. ClustalX alignments highlighting the relatively weak alignment of between mouse Claudin 13 (AY554386) and Fugu Claudin 13 (AF516681). B. Contrasting strong alignments between mouse Claudin 3 (NP_034032) and Fugu Claudin 3 (AAT64047). Combined with phylogenetic evidence this indicates that the suggested Fugu Claudin 13 is unlikely to be the true ortholog of mouse Claudin 13. (0.53 MB TIF) [file pone.0012667.s003.tif]

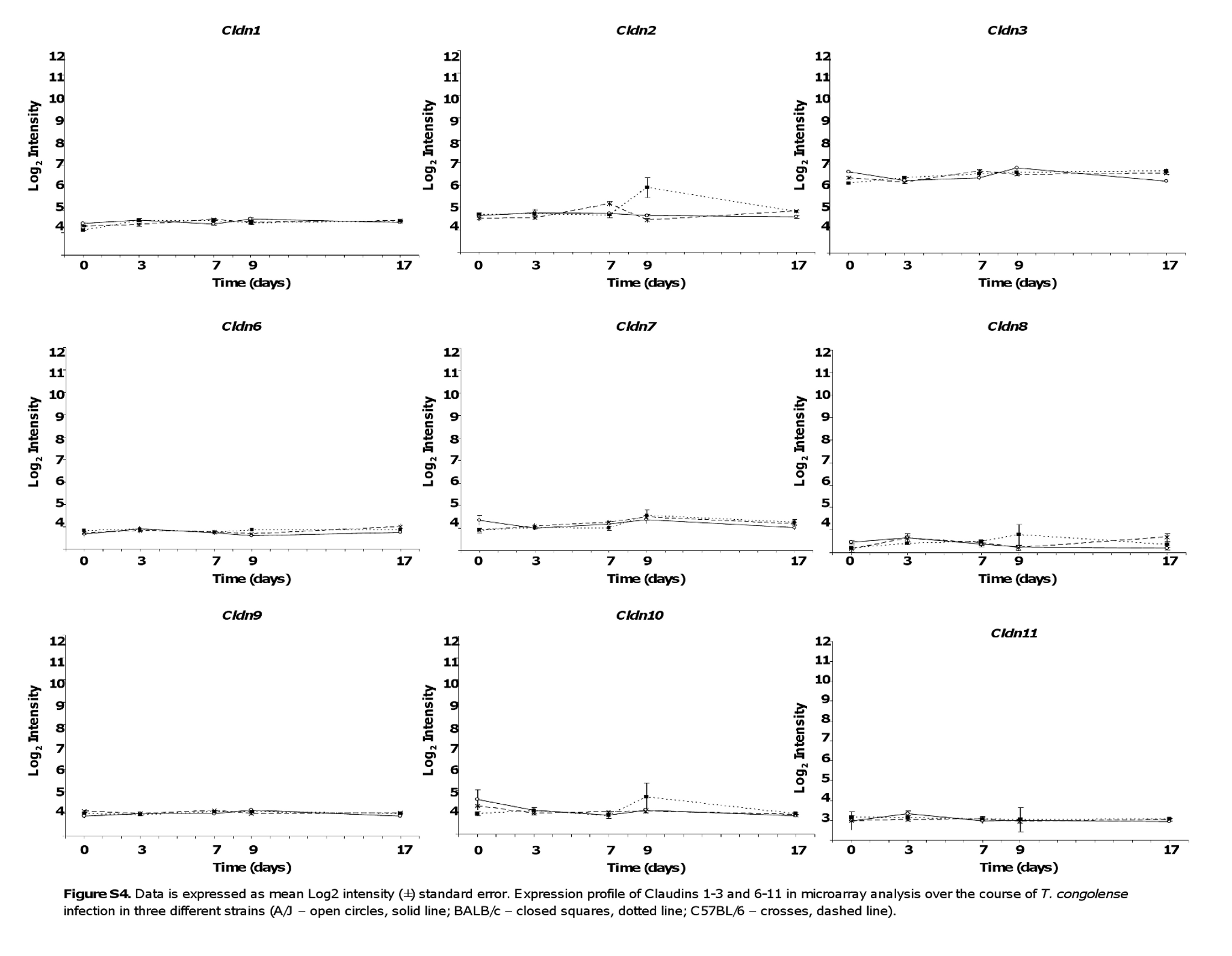

Supplement: Figure S4 — Data is expressed as mean Log2 intensity (± standard error. Expression profiles of Claudins 1–3 and 6–11 in microarray analysis over the course of T. congolense infection in three different strains (A/J - open circles, solid line; BALB/c - closed squares, dotted line; C57BL/6 - crosses, dashed line). (0.34 MB TIF) [file pone.0012667.s004.tif]

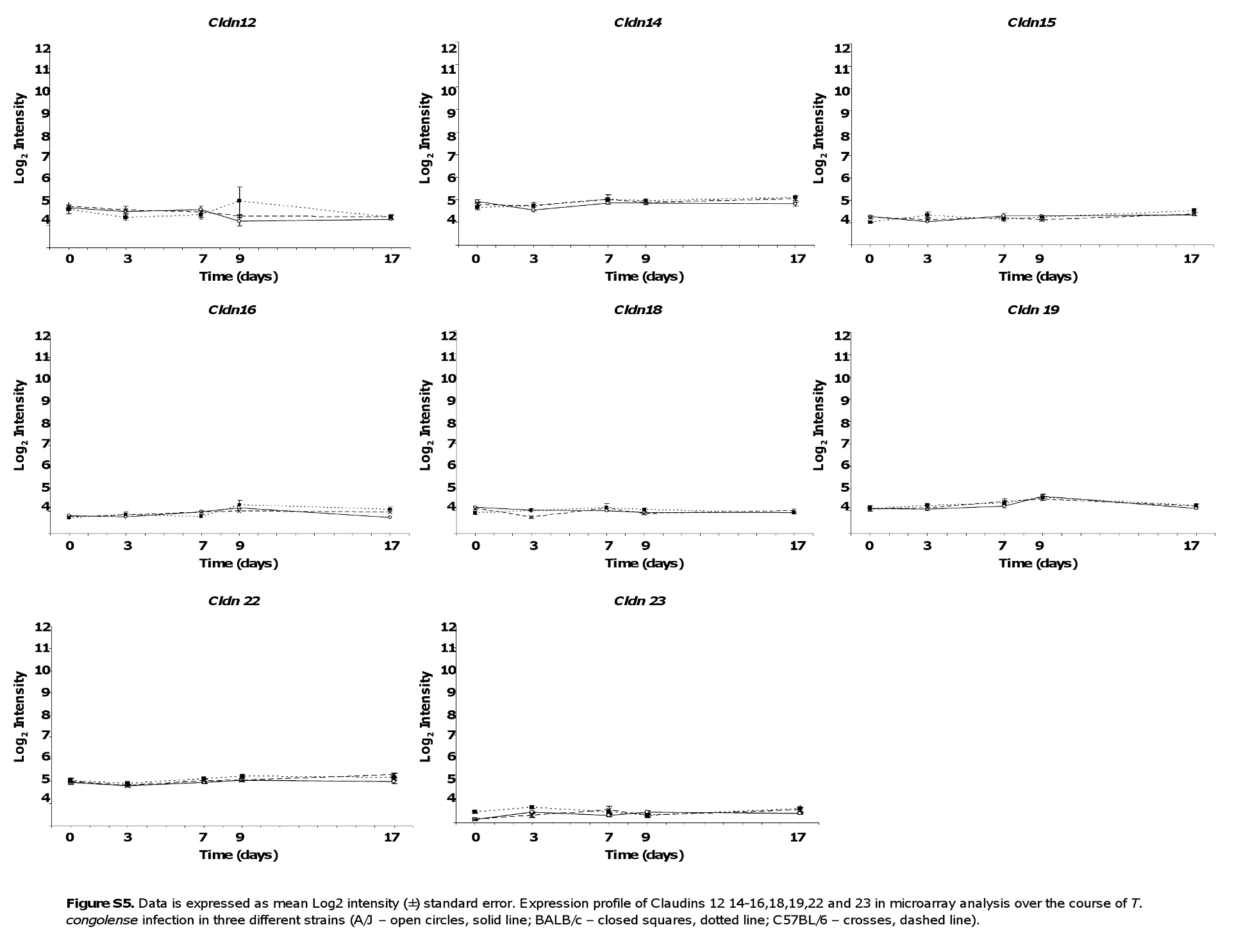

Supplement: Figure S5 — Data is expressed as mean Log2 intensity (± standard error. Expression profiles of Claudins 12, 14–16, 18, 19, 22 and 23 in microarray analysis over the course of T. congolense infection in three different strains (A/J - open circles, solid line; BALB/c - closed squares, dotted line; C57BL/6 - crosses, dashed line). (0.22 MB TIF) [file pone.0012667.s005.tif]

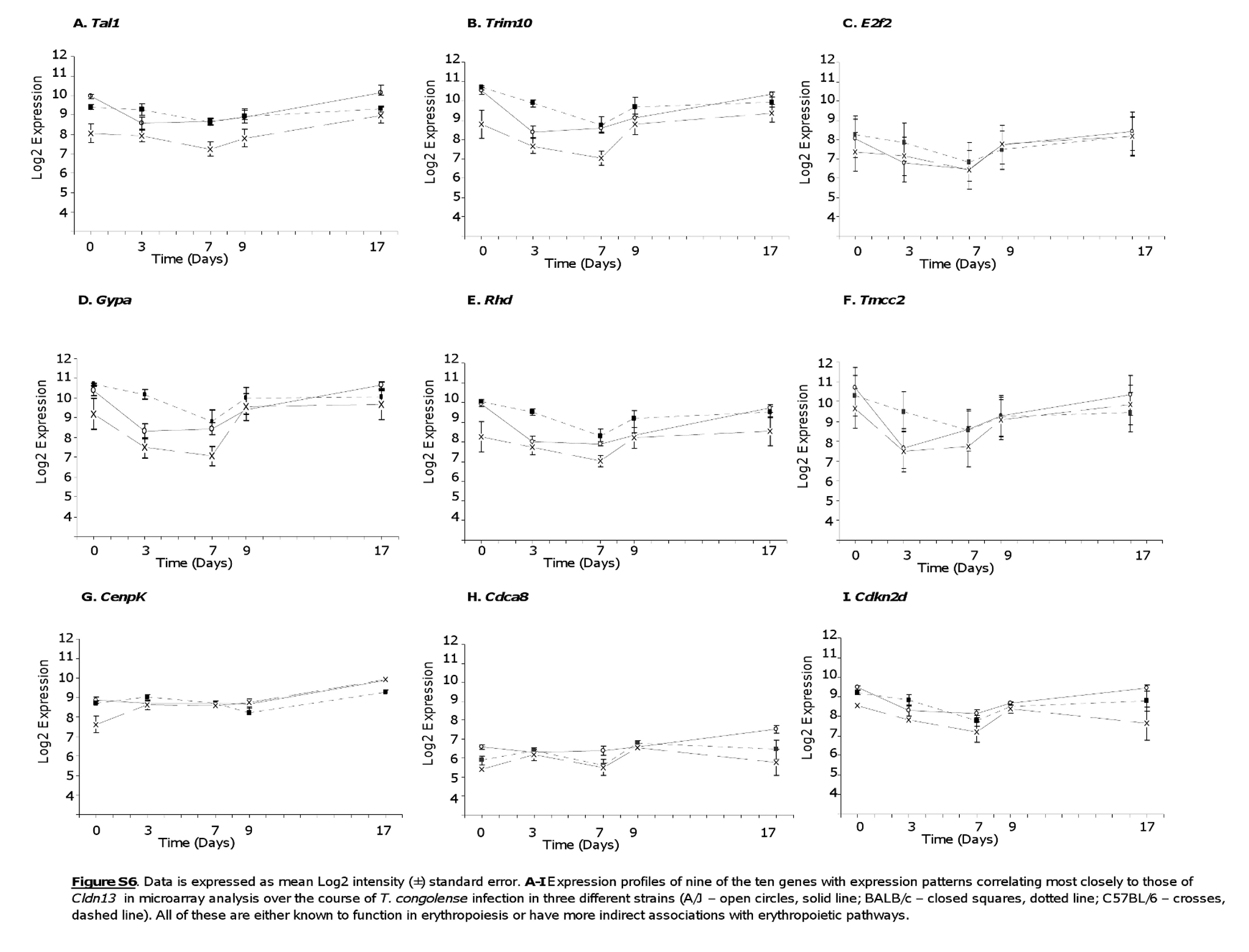

Supplement: Figure S6 — Data is expressed as mean Log2 intensity (±) standard error. A–I: Expression profiles of nine of the ten genes with expression patterns correlating most closely to those of Cldn13 in microarray analysis of T. congolense infection in three different strains (A/J - open circles, solid line; BALB/c - closed squares, dotted line; C57BL/6 - crosses, dashed line). All of these are either known to function in erythropoiesis or have more indirect associations with erythropoietic pathways. (0.43 MB TIF) [file pone.0012667.s006.tif]
