# Supplementary material for: Claudin 13, a Member of the Claudin Family Regulated in Mouse Stress Induced Erythropoiesis
Source: PLoS One. 2010 Sep 10;5(9):e12667. doi: 10.1371/journal.pone.0012667 (PMC2937028; doi:10.1371/journal.pone.0012667)
Supplement: Table S1 — (0.03 MB DOC) [file pone.0012667.s007.doc]

| **Exon** | **Includes** | **cDNA**  **position** | **5’ splice site** | **Exon size**  **(bp)** | **3’ splice site** | **Intron size (bp)** |
| --- | --- | --- | --- | --- | --- | --- |
| 1, 5’ UTR | ATG (198) | 1 - 825 | *ttcaccagat* AGGGT | 825 | CTTAG **gt**aagtgtct | 211 |
| 2, 3’UTR | TGA (831)  PolyA (1045) | 826 - 1067 | tattctac**ag** ATGTT | 241 | CCTAG *actggact* | - - |

**Table S1. Genomic structure of *Mus musculus Cldn13***

Intron and exon sizes are given in addition to the relative position within the cDNA sequence (AF516681). Coding sequence is in capitals. Bold represents intronic donor and acceptor splice junctions. Italics indicate genomic sequence.
