# Supplementary material for: Claudin 13, a Member of the Claudin Family Regulated in Mouse Stress Induced Erythropoiesis
Source: PLoS One. 2010 Sep 10;5(9):e12667. doi: 10.1371/journal.pone.0012667 (PMC2937028; doi:10.1371/journal.pone.0012667)
Supplement: Table S2 — (0.03 MB DOC) [file pone.0012667.s008.doc]

| **Predicted Motif** | **Amino Acid position (s)** |
| --- | --- |
| N-glycosylation sites | 109–112, 206–209 |
| Protein kinase C phosphorylation sites | 154–156, 198–200 |
| Casein kinase II phosphorylation sites | 4–7, 33–36, 39–42, 44–47, 64–67, 111–114 |
| N-myristoylation sites | 20–25, 89–94, 93–98, 122–127, 160–165, 177–182 |
| Prokaryotic membrane lipoprotein lipid attachment site  (SLGWVGAIVSC, PDOC00013). | 15-25 |

**Table S2. Predicted functional CLDN13 protein motifs.**
