# Supplementary material for: Claudin 13, a Member of the Claudin Family Regulated in Mouse Stress Induced Erythropoiesis
Source: PLoS One. 2010 Sep 10;5(9):e12667. doi: 10.1371/journal.pone.0012667 (PMC2937028; doi:10.1371/journal.pone.0012667)
Supplement: Table S3 — (0.12 MB DOC) [file pone.0012667.s009.doc]

| **Abbreviation** | **Claudin Name** | **Accession** | **Chromo.** | **Species** |
| --- | --- | --- | --- | --- |
| HS_CLDN1 | Claudin 1 | NP_066924 | 3 | *Homo sapiens* |
| HS_CLDN2 | Claudin 2 | NP_065117 | X | *Homo sapiens* |
| HS_CLDN3 | Claudin 3 | NP_001297 | 7 | *Homo sapiens* |
| HS_CLDN4 | Claudin 4 | NP_001296 | 7 | *Homo sapiens* |
| HS_CLDN5 | Claudin 5 | NP_003268 | 22 | *Homo sapiens* |
| HS_CLDN6 | Claudin 6 | NP_067018 | 16 | *Homo sapiens* |
| HS_CLDN7 | Claudin 7 | NP_001298 | 17 | *Homo sapiens* |
| HS_CLDN8 | Claudin 8 | NP_955360 | 21 | *Homo sapiens* |
| HS_CLDN9 | Claudin 9 | NP_066192 | 16 | *Homo sapiens* |
| HS_CLDN10b | Claudin 10 isoform b | NP_008915 | 13 | *Homo sapiens* |
| HS_CLDN11 | Claudin 11 | NP_005593 | 3 | *Homo sapiens* |
| HS_CLDN12 | Claudin 12 | NP_036261 | 7 | *Homo sapiens* |
| HS_CLDN14 | Claudin 14 | NP_036262 | 21 | *Homo sapiens* |
| HS_CLDN15a | Claudin 15 isoform 1 | NP_055158 | 7 | *Homo sapiens* |
| HS_CLDN16 | Claudin 16 | NP_006571 | 3 | *Homo sapiens* |
| HS_CLDN17 | Claudin 17 | NP_036263 | 21 | *Homo sapiens* |
| HS_CLDN18a | Claudin 18 isoform 1 | NP_057453 | 3 | *Homo sapiens* |
| HS_CLDN19 | Claudin 19 | NP_683763 | 1 | *Homo sapiens* |
| HS_CLDN20 | Claudin 20 | NP_001001346 | 6 | *Homo sapiens* |
| HS_CLDN22 | Claudin 22 | XP_931889 | 4 | *Homo sapiens* |
| HS_CLDN23 | Claudin 23 | NP_919260 | 8 | *Homo sapiens* |
| MM_CLDN1 | Claudin 1 | NP_057883 | 16 | *Mus musculus* |
| MM_CLDN2 | Claudin 2 | NP_057884 | X | *Mus musculus* |
| MM_CLDN3 | Claudin 3 | NP_034032 | 5 | *Mus musculus* |
| MM_CLDN4 | Claudin 4 | NP_034033 | 5 | *Mus musculus* |
| MM_CLDN5 | Claudin 5 | NP_038833 | 16 | *Mus musculus* |
| MM_CLDN6 | Claudin 6 | NP_061247 | 17 | *Mus musculus* |
| MM_CLDN7 | Claudin 7 | NP_058583 | 11 | *Mus musculus* |
| MM_CLDN8 | Claudin 8 | NP_061248 | 16 | *Mus musculus* |
| MM_CLDN9 | Claudin 9 | NP_064689 | 17 | *Mus musculus* |
| MM_CLDN10b | Claudin 10 isoform b | NP_067361 | 14 | *Mus musculus* |
| MM_CLDN11 | Claudin 11 | NP_032796 | 3 | *Mus musculus* |
| MM_CLDN12 | Claudin 12 | NP_075028 | 5 | *Mus musculus* |
| MM_CLDN13 | Claudin 13 | AAN03863 | 5 | *Mus musculus* |
| MM_CLDN14 | Claudin 14 | NP_062373 | 16 | *Mus musculus* |
| MM_CLDN15 | Claudin 15 | NP_068365 | 5 | *Mus musculus* |
| MM_CLDN16 | Claudin 16 | NP_444471 | 16 | *Mus musculus* |
| MM_CLDN17 | Claudin 17 | NP_852467 | 16 | *Mus musculus* |
| MM_CLDN18 | Claudin 18 | NP_062789 | 9 | *Mus musculus* |
| MM_CLDN19a | Claudin 19 isoform 1 | NP_001033679 | 4 | *Mus musculus* |
| MM_CLDN22 | Claudin 22 | Q9D7U6 | 8 | *Mus musculus* |
| MM_CLDN23 | Claudin 23 | NP_082274 | 8 | *Mus musculus* |
| DR_CLDN5 | Claudin 5 | NP_998439 | 20 | *Danio rerio* |
| DR_CLDN7 | Claudin 7 | NP_571712 | 10 | *Danio rerio* |
| DR_CLDN10 | Claudin 10 | NP_001007038 | 6 | *Danio rerio* |
| DR_CLDN11 | Claudin 11b | NP_571847 | 24 | *Danio rerio* |
| DR_CLDN12 | Claudin 12 | NP_571848 | 9 | *Danio rerio* |
| DR_CLDN19 | Claudin 19 (aka Claudin 1) | NP_571845 | Unplaced | *Danio rerio* |
| DR_CLDNa | Claudin a | NP_571837 | 1 | *Danio rerio* |
| DR_CLDNb | Claudin b | NP_571838 | 15 | *Danio rerio* |
| DR_CLDNc | Claudin c | NP_571839 | 15 | *Danio rerio* |
| DR_CLDNd | Claudin d | NP_851295 | 21 | *Danio rerio* |
| DR_CLDNe | Claudin e | NP_571840 | 15 | *Danio rerio* |
| DR_CLDNf | Claudin f | NP_571841 | 1 | *Danio rerio* |
| DR_CLDNg | Claudin g | NP_851296 | 24 | *Danio rerio* |
| DR_CLDNh | Claudin h | NP_571842 | 6 | *Danio rerio* |
| DR_CLDNi | Claudin i | NP_571843 | 3 | *Danio rerio* |
| DR_CLDNj | Claudin j | NP_571844 | 15 & 5 | *Danio rerio* |
| TR_CLDN1 | Claudin 1 | AAT64078 | -- | *Takifugu rubripes* |
| TR_CLDN2 | Claudin 2 | AAT64079 | -- | *Takifugu rubripes* |
| TR_CLDN3d | Claudin 3d | AAT64058 | -- | *Takifugu rubripes* |
| TR_CLDN5c | Claudin 5c | AAT64049 | -- | *Takifugu rubripes* |
| TR_CLDN6 | Claudin 6 | AAT64055 | -- | *Takifugu rubripes* |
| TR_CLDN7a | Claudin 7a | AAT64069 | -- | *Takifugu rubripes* |
| TR_CLDN8d | Claudin 8d | AAT64046 | -- | *Takifugu rubripes* |
| TR_CLDN10e | Claudin 10e | AAT64042 | -- | *Takifugu rubripes* |
| TR_CLDN11a | Claudin 11a | AAT64084 | -- | *Takifugu rubripes* |
| TR_CLDN12 | Claudin 12 | AAT64072 | -- | *Takifugu rubripes* |
| TR_CLDN13 | Claudin 13 | AAT64056 | -- | *Takifugu rubripes* |
| TR_CLDN14b | Claudin 14b | AAT64039 | -- | *Takifugu rubripes* |
| TR_CLDN15b | Claudin 15b | AAT64083 | -- | *Takifugu rubripes* |
| TR_CLDN18 | Claudin 18 | AAT64070 | -- | *Takifugu rubripes* |
| TR_CLDN19 | Claudin 19 | AAT64088 | -- | *Takifugu rubripes* |
| TR_CLDN20b | Claudin 20b | AAT64080 | -- | *Takifugu rubripes* |
| TR_CLDN23a | Claudin 23a | AAT64074 | -- | *Takifugu rubripes* |
| TR_CLDN25 | Claudin 25 | AAT64090 | -- | *Takifugu rubripes* |
| TR_CLDN26 | Claudin 26 | AAT64089 | -- | *Takifugu rubripes* |
| TR_CLDN27d | Claudin 27d | AAT64064 | -- | *Takifugu rubripes* |
| TR_CLDN28a | Claudin 28a | AAT64053 | -- | *Takifugu rubripes* |
| TR_CLDN29a | Claudin 29a | AAT64062 | -- | *Takifugu rubripes* |
| TR_CLDN30d | Claudin 30d | AAT64066 | -- | *Takifugu rubripes* |
| TR_CLDN31 | Claudin 31 | AAT64077 | -- | *Takifugu rubripes* |
| TR_CLDN32b | Claudin 32b | AAT64082 | -- | *Takifugu rubripes* |
| TR_CLDN33c | Claudin 33c | AAT64068 | -- | *Takifugu rubripes* |
| CI_283850 | estExt_fgenesh3_pm.C_chr_08q0113 | Cioin2:283850 | 8 | *Ciona intestinalis* |
| CI_254728 | gw1.10q.129.1 | Cioin2:254728 | 10 | *Ciona intestinalis* |
| CI_254477 | gw1.10q.123.1 | Cioin2:254477 | 10 | *Ciona intestinalis* |
| CI_257297 | gw1.01q.501.1 | Cioin2:257297 | 1 | *Ciona intestinalis* |
| CI_241221 | estExt_genewise1.C_chr_05q1042 | Cioin2:241221 | 5 | *Ciona intestinalis* |

**Table S3. Claudin family sequence dataset.**

Sequences used to generate the phylogenetic relationships between CLDN13 and other Claudin proteins. The abbreviations in the left hand column correspond to sequence identifiers within the phylogenetic trees (Figure 4). Chromosome location as reported by NCBI MapViewer (June 2010). Unknown chromosomal location is shown by (--).
