# Supplementary material for: Claudin 13, a Member of the Claudin Family Regulated in Mouse Stress Induced Erythropoiesis
Source: PLoS One. 2010 Sep 10;5(9):e12667. doi: 10.1371/journal.pone.0012667 (PMC2937028; doi:10.1371/journal.pone.0012667)
Supplement: Table S4 — (0.03 MB DOC) [file pone.0012667.s010.doc]

| **Gene** | **Forward primer** | **Reverse Primer** |
| --- | --- | --- |
| **beta actin (northern/ RT-PCR)** | GTATGGAATCCTGTGGCATCC | CGTACTCCTGCTTGCTGATCC |
| ***Cldn3* (RT-PCR)** | GCCAACACCATCATCAGGGATTTC | GCAGGAGCAACAGCAAGG |
| ***Cldn4* (RT-PCR)** | CCTTCCGTTGATTAGCAATGACTC | AATCCACCTCCACCCTTCTTCC |
| ***Cldn13* (RT-PCR)** | ATGGTCGTCAGCAAACAAGAGG | TCAAACATCTAAGGTATCGTTG |
| ***Cldn13* (northern probe)** | TGGGAGGCATCCTGCTCTGTGTC | GGGTCCCTTAGATAGGAGACTCG |

**Table S4. Oligonucleotide primers used in this study.**
